# Effects of a sensorimotor training program on balance and knee motor control in male handball players with hearing impairment: a randomized controlled trial

**DOI:** 10.3389/fspor.2026.1812503

**Published:** 2026-07-29

**Authors:** Goran Bobić, Tatjana Trošt, Iris Zavoreo

**Affiliations:** 1 University of Zagreb, Faculty of Kinesiology, Zagreb, Croatia; 2 Klinika za neurologiju (Clinic of Neurology), Sestre Milosrdnice University Hospital Center, Zagreb, Croatia

**Keywords:** dynamic balance, handball, hearing impairment, knee joint position sense, motor control, proprioception, randomized controlled trial, sensorimotor training

## Abstract

The ability to maintain balance may be reduced in individuals with hearing impairment compared to healthy populations, potentially increasing injury risk in sports contexts. The aim of this study was to examine the effects of a sensorimotor training program on balance and knee motor control in male handball players with hearing impairment. A randomized controlled trial was conducted with 24 male handball players with hearing impairment (28 ± 6.3 years), randomly assigned to an experimental group (*n* = 12) and a control group (*n* = 12). The experimental group completed a five-week sensorimotor training program (20 sessions), while the control group continued regular training. Dynamic balance was assessed using the Star Excursion Balance Test, while knee motor control was evaluated using joint position sense and a sport-specific agility test. Between-group and within-group differences were analysed using repeated-measures ANOVA and paired t-tests, with effect sizes calculated using Cohen's d. Results indicate improvements in dynamic balance and knee joint position sense in the experimental group, while no significant improvements were observed in agility performance. These findings suggest that sensorimotor training may improve selected components of motor control in athletes with hearing impairment.

## 1 Introduction

Handball is a high-intensity team sport requiring complex motor, technical, and tactical abilities (1, 2). Its growing global participation includes athletes with disabilities, including those with hearing impairment. Athletes with hearing impairment may demonstrate altered balance control due to reduced auditory input, which contributes to multisensory integration in postural regulation (3, 4). Previous findings are mixed, reporting both impaired and comparable balance performance relative to hearing individuals, suggesting compensatory sensory mechanisms such as sensory reweighting (5, 6). Deficits in balance and motor control may increase injury risk in handball players (7, 8). Sensorimotor training has been shown to improve neuromuscular control and balance in athletic populations, particularly in team sports such as handball (8–11); however, evidence in athletes with hearing impairment remains limited (12, 13). In addition, sensorimotor and proprioceptive training are considered key components in promoting neuroplastic adaptations within sensorimotor networks, particularly in populations relying more heavily on somatosensory input for postural regulation (14–16). In adapted sports contexts, structured neuromuscular training may therefore play a compensatory role in maintaining motor stability under sensory constraints (3, 10, 17).

Modern handball performance is characterized by repeated high-intensity actions such as accelerations, decelerations, jumps, landings, and rapid changes of direction performed under time pressure and physical contact (18, 19). These actions place substantial demands on the neuromuscular system, particularly on the lower extremities, where efficient motor control of the knee joint is essential for both performance and injury prevention. Agility, defined as the ability to rapidly change direction while maintaining postural control and movement efficiency, represents a key neuromuscular performance component in handball and is strongly associated with injury risk and performance capacity in multidirectional sports (18, 20). Injury prevention has become a central component of contemporary sports training (21). Neuromuscular training interventions have been shown to reduce the incidence of lower-limb injuries, particularly non-contact knee injuries in team sports, by improving movement control and landing mechanics (11, 22). Similar approaches have been successfully applied across different sport disciplines such as football, basketball, and volleyball, demonstrating improvements in dynamic stability and change-of-direction performance (23). Within this framework, sensorimotor training is widely recognized for enhancing proprioception, neuromuscular coordination, and dynamic joint stability (24, 25). Postural control is a complex function based on the integration of visual, vestibular, somatosensory, and auditory inputs. Although auditory information is not traditionally considered a primary contributor to balance, recent evidence suggests that auditory cues may influence spatial orientation and postural adjustments through audio-vestibular interactions (26, 27). Consequently, hearing impairment may induce adaptive reorganization of postural control strategies. Individuals with hearing impairment often show altered balance performance compared to hearing individuals; however, findings remain inconsistent, with both deficits and compensatory adaptations reported (28–30). In handball, these adaptations may be further challenged due to the highly dynamic and unpredictable nature of match play, where rapid postural adjustments are required under fatigue and physical contact. Lower-limb injuries, particularly involving the knee joint, represent a substantial proportion of handball-related injuries and commonly occur during high-intensity movements such as jumping, landing, and directional changes (31). This highlights the importance of optimizing neuromuscular control strategies to enhance both performance and injury resilience. Sensorimotor training is proposed to improve neuromuscular control through enhanced sensory integration and more efficient motor output. It is also associated with neuroplastic adaptations within central sensorimotor pathways, resulting in improved joint stability and movement coordination (24, 25). Although evidence supports its effectiveness in healthy and athletic populations, research in athletes with hearing impairment remains limited, particularly regarding sport-specific performance variables such as knee joint motor control, dynamic balance, and agility. Therefore, there is a clear need to investigate the effects of sensorimotor training on a comprehensive set of neuromuscular performance outcomes in athletes with hearing impairment participating in handball.

The aim of this study was to examine the effects of a sensorimotor training program on knee joint motor control, dynamic balance, and agility in male handball players with hearing impairment. It was hypothesized that sensorimotor training would result in statistically significant improvements in knee joint motor control, dynamic balance, and agility in male handball players with hearing impairment. Specifically, it was expected that participants would demonstrate enhanced knee joint motor control following the intervention, accompanied by improvements in dynamic balance performance and faster, more efficient changes of direction reflecting improved agility. It is expected that sensorimotor training will enhance neuromuscular efficiency through improved sensory integration, proprioceptive accuracy, and motor coordination, leading to improved postural stability, knee joint control, and agility performance under sport-specific conditions.

## 2 Materials and methods

This study was designed as a randomized controlled trial with two parallel groups (experimental and control group). The experimental group completed a five-week sensorimotor training program, while the control group continued their regular training activities. The study followed a quantitative experimental design framework commonly used in sports performance analysis research (32, 33). The aim was to examine the effects of sensorimotor training on dynamic balance and knee motor control in male handball players with hearing impairment.

### 2.1 Participants

The twenty-four male handball players with hearing impairment who met the inclusion criteria participated in the study (28 ± 6.3 years). Inclusion criteria included diagnosed hearing impairment (≥55 dB in the better ear), age under 40 years, and regular participation in handball training. Exclusion criteria included recent lower limb injury, neuromuscular disorders, and chronic pain conditions. All participants were active competitive handball players. Participants were randomly assigned to either the experimental group (*n* = 12) or the control group (*n* = 12) using a computer-generated randomization sequence created with an online tool (https://www.random.org). Allocation was performed in a 1:1 ratio after completion of baseline assessments by the principal investigator. All participants were members of the same competitive handball team and followed an identical, coach-prescribed weekly training schedule; therefore, the reported weekly training volume (4.5 h per week) reflects scheduled team training exposure, with no between-participant variation in planned training duration during the study period. Due to the limited population of elite male handball players with hearing impairment available in the region, participant recruitment was restricted to all eligible athletes who met the predefined inclusion criteria during the recruitment period. Consequently, no stratified or blocked randomization was applied. Participant demographic and anthropometric characteristics at baseline are presented in Table 1. No significant baseline differences were observed between the experimental and control groups for age, body height, body mass, body mass index, or weekly training volume (all *p* > 0.05). Due to the nature of the intervention, blinding of participants, coaches, and assessors was not feasible. To reduce the influence of visual input during balance and proprioception testing, participants wore an eye-covering band throughout all measurement procedures. All participants completed the study protocol and no drop-outs occurred. The study was conducted in accordance with the Declaration of Helsinki. All participants provided written informed consent prior to participation.

**Table 1:** Baseline demographic and anthropometric characteristics of participants.

Variable	Experimental group	Control group	*p*
Age (years)	27.10 ± 7.13	28.20 ± 5.47	0.70
Height (m)	1.82 ± 0.07	1.80 ± 0.05	0.41
Body mass (kg)	79.20 ± 7.44	78.60 ± 5.27	0.84
Body mass index (kg/m^2^)	23.82 ± 1.28	24.24 ± 0.83	0.39
Weekly training volume (h/week)	4.50 ± 0.00	4.50 ± 0.00	1.00

Values are presented as mean ± standard deviation (SD). Between-group comparisons were performed using independent-samples *t*-tests. Statistical significance was set at *p* < 0.05.

### 2.2 Procedures

Dynamic balance was assessed using the Star Excursion Balance Test (SEBT), a validated and reliable tool for assessment of lower-limb dynamic postural control (34). Participants performed reach tasks in multiple directions while maintaining single-leg stance. Knee motor control was assessed using joint position sense testing based on the ability to reproduce predefined knee flexion angles (15°, 60°, and 105°). This method is widely used in proprioception research and reflects sensorimotor system function in the absence of visual feedback (14, 16, 35). Agility performance was evaluated using the SMSS test (speed of movement in a sport-specific defensive sliding task), which includes handball-specific change-of-direction and lateral movement patterns (1, 2). The test was performed separately in the preferred and non-preferred movement directions and was used as an indicator of sport-specific agility and movement efficiency.

### 2.3 Sensorimotor training

The experimental group completed a five-week sensorimotor training program consisting of 20 sessions (4 sessions per week). The training program included balance exercises, plyometric drills, change-of-direction tasks and sport-specific handball movements. The program was based on established neuromuscular and proprioceptive training principles used in injury prevention and performance enhancement literature (8–10, 36, 37). Training intensity and complexity were progressively increased throughout the intervention.

### 2.4 Statistical analyses

Data were expressed as mean ± standard deviation. The Shapiro–Wilk test was used to assess normality of data distribution (Supplementary Table S1). The results did not indicate meaningful departures from normality across variables and groups (all *p* > 0.05), supporting the use of parametric statistical procedures. Homogeneity of variance was evaluated using Levene's test applied to pre–post change scores between groups, as this approach allows assessment of equality of variance in intervention-related change rather than baseline dispersion (Supplementary Table S2). Although violations of homogeneity of variance were observed for several variables, repeated-measures ANOVA was retained due to the equal sample sizes between groups (*n* = 12 per group), as this design is generally robust to moderate violations of variance homogeneity under balanced conditions. The assumption of sphericity was not a concern due to the two-level within-subject factor (pre–post design). Within-group differences between pre- and post-intervention measurements were analysed using paired-samples t-tests. Between-group differences over time were examined using repeated-measures analysis of variance (ANOVA) with group ×  time interaction effects. The group ×  time interaction was considered the primary indicator of intervention effectiveness. Paired-samples t-tests were performed as complementary within-group (*post hoc*) analyses to describe pre–post changes within each group and should not be interpreted as the primary evidence of training efficacy. Effect sizes for pre–post changes were calculated using Cohen's d to provide a standardized measure of within-group change that is easily interpretable and commonly reported in applied sports science research. Although partial eta squared is often used to report effect sizes for ANOVA-based omnibus effects, Cohen's d was used to describe the magnitude of pre–post changes within each group, which was of primary practical interest in this intervention study. Effect sizes were interpreted as follows: trivial (<0.20), small (0.20–0.49), moderate (0.50–0.79), and large (≥0.80). A *post hoc* statistical power analysis was performed using G*Power (version 3.1; Heinrich Heine University Düsseldorf, Germany). The achieved power (1 − *β*) was 0.65 for the primary group ×  time interaction effect, based on the observed effect sizes and the total sample size (*N* = 24). Data were initially organized and processed using Microsoft Excel (Microsoft Corp., Redmond, WA, USA). Statistical analyses were performed using STATISTICA (version 13; TIBCO Software Inc., Palo Alto, CA, USA). Statistical significance was set at *p* < 0.05.

## 3 Results

### 3.1 Participant characteristics (body mass and BMI)

No statistically significant changes in body mass or body mass index (BMI) were observed in either group over the 5-week intervention period. In the experimental group, body mass showed a non-significant decrease from 79.20 ± 7.44 kg to 78.40 ± 7.38 kg (*p* = 0.07), while BMI decreased from 23.82 ± 1.28 to 23.58 ± 1.40 (*p* = 0.08). In the control group, body mass remained stable (78.60 ± 5.27 kg vs. 78.80 ± 5.85 kg; *p* = 0.74), as did BMI (24.24 ± 0.83 vs. 24.29 ± 1.02; *p* = 0.76). No between-group differences were observed at post-test for either body mass (*p* = 0.89) or BMI (*p* = 0.21).

### 3.2 Motor control outcomes (within-group *post hoc* analyses)

Descriptive statistics for motor control outcomes are presented in Table 2, whereas within-group *post hoc* comparisons are shown in Supplementary Table S3. Within-group comparisons (paired-samples t-tests) indicated improvements in selected motor control variables in the experimental group, whereas no meaningful changes were observed in the control group. In the experimental group, significant improvements were observed in knee joint position sense at 60° flexion for both the preferred limb (*p* = 0.05, *d* = 0.73) and non-preferred limb (*p* = 0.05, *d* = 0.78), indicating moderate-to-large effects of the intervention. No statistically significant changes were found for SMSS performance or for knee joint position sense at 15° and 105° flexion (all *p* > 0.05), although small effect sizes (*d* = 0.08–0.35) suggested minor improvements in several variables. In the control group, no statistically significant pre–post changes were observed across any motor control variable (all *p* > 0.05), with trivial to small effect sizes (*d* = 0.00–0.23).

**Table 2:** Descriptive statistics for motor control outcomes (mean ± SD).

Variable	Group	Pre-test (mean ± SD)	Post-test (mean ± SD)
SMSS (*p*)	E	7.54 ± 0.69	7.39 ± 0.61
SMSS (*p*)	C	7.54 ± 0.66	7.68 ± 0.69
SMSS (*n*)	E	7.60 ± 0.71	7.45 ± 0.70
SMSS (*n*)	C	7.76 ± 0.78	7.69 ± 0.80
Ō 15° (*p*)	E	4.72 ± 2.31	3.92 ± 2.25
Ō 15° (*p*)	C	5.20 ± 3.38	5.88 ± 3.29
Ō 60° (*p*)	E	7.69 ± 4.61	4.32 ± 1.29
Ō 60° (*p*)	C	5.54 ± 3.96	5.53 ± 3.97
Ō 105° (*p*)	E	8.66 ± 5.21	7.72 ± 5.09
Ō 105° (*p*)	C	7.47 ± 3.45	6.74 ± 3.62
Ō 15° (*n*)	E	4.64 ± 2.61	4.42 ± 2.59
Ō 15° (*n*)	C	5.11 ± 3.01	4.42 ± 2.67
Ō 60° (*n*)	E	6.95 ± 4.07	3.78 ± 1.95
Ō 60° (*n*)	C	5.72 ± 3.95	5.76 ± 3.89
Ō 105° (*n*)	E	9.97 ± 4.15	9.57 ± 4.97
Ō 105° (*n*)	C	8.13 ± 4.46	8.16 ± 4.42

Values are presented as mean ± SD.

E, experimental group; C, control group; p, preferred limb; n, non-preferred limb; SMSS, speed of movement in defensive sliding task; Ō, absolute angular error in knee joint position sense.

### 3.3 Motor control outcomes (between-group effects)

Repeated-measures ANOVA (group ×  time interaction; Table 3) revealed significant between-group differences for knee joint position sense at 60° flexion in both the preferred limb (F = 5.363, *p* = 0.033, *d* = 0.73) and non-preferred limb (F = 4.755, *p* = 0.043, *d* = 0.78), indicating greater improvements in the experimental group compared to controls. No significant group ×  time interactions were found for SMSS performance or for knee joint position sense at 15° and 105° flexion (all *p* > 0.05). The SMSS preferred limb showed a non-significant trend toward interaction (*p* = 0.051), which did not meet the predefined level of statistical significance.

**Table 3:** Group × time interaction effects for motor control outcomes (repeated-measures ANOVA).

Variable	*F*	*p*	Cohen's d
SMSS (*p*)	4.363	0.051	0.22
SMSS (*n*)	0.553	0.467	0.21
Ō 15° (*p*)	3.373	0.083	0.35
Ō 60° (*p*)	5.363	0.033	0.73
Ō 105° (*p*)	0.006	0.937	0.18
Ō 15° (*n*)	0.307	0.587	0.08
Ō 60° (*n*)	4.755	0.043	0.78
Ō 105° (*n*)	0.045	0.835	0.10

SMSS, speed of movement in defensive sliding task; Ō, absolute angular error in knee joint position sense. ES represents Cohen's d calculated for pre–post changes in the experimental group. Values represent group × time interaction effects from repeated-measures ANOVA. *p*, preferred limb; *n*, non-preferred limb. Statistical significance was set at *p* < 0.05.

### 3.4 Dynamic balance (within-group *post hoc* analyses)

Descriptive statistics for dynamic balance variables obtained from the Star Excursion Balance Test are presented in Table 4, while within-group *post hoc* comparisons are presented in Supplementary Table S4. Within-group analysis (paired-samples t-tests) demonstrated consistent improvements in dynamic balance following the intervention in the experimental group. Significant pre–post improvements were observed in multiple Star Excursion Balance Test (STAR) variables, particularly in the posteromedial direction (preferred limb: *p* = 0.02, *d* = 0.81; non-preferred limb: *p* = 0.04, *d* = 0.57), posterolateral direction (preferred limb: *p* = 0.04, *d* = 0.43; non-preferred limb: *p* = 0.05, *d* = 0.42), and total reach distance (preferred limb: *p* = 0.02, *d* = 0.40; non-preferred limb: *p* = 0.05, *d* = 0.48). Effect sizes ranged from small to large (*d* = 0.18–0.81). In contrast, the control group showed no statistically significant changes in any STAR variable (all *p* > 0.05), with negligible effect sizes.

**Table 4:** Descriptive statistics for dynamic balance outcomes (Star Excursion Balance Test; mean ± SD).

Variable	Group	Pre-test (mean ± SD)	Post-test (mean ± SD)
STAR A (*p*)	E	80.30 ± 14.59	84.60 ± 10.41
	C	82.23 ± 13.68	82.33 ± 13.02
STAR AM (*p*)	E	82.47 ± 9.79	83.83 ± 10.21
	C	81.67 ± 9.71	81.63 ± 8.50
STAR M (*p*)	E	84.17 ± 11.35	90.20 ± 15.57
	C	83.67 ± 11.05	83.43 ± 10.94
STAR PM (*p*)	E	83.33 ± 10.90	92.20 ± 14.82
	C	83.57 ± 12.85	83.07 ± 12.88
STAR P (*p*)	E	86.33 ± 17.73	91.60 ± 16.21
	C	82.63 ± 15.95	82.23 ± 16.44
STAR PL (*p*)	E	79.33 ± 16.62	86.40 ± 12.96
	C	80.00 ± 17.67	80.47 ± 17.95
STAR L (*p*)	E	68.27 ± 16.04	73.30 ± 13.39
	C	68.57 ± 15.44	68.60 ± 14.46
STAR AL (*p*)	E	74.80 ± 11.47	76.83 ± 10.29
	C	75.17 ± 11.26	75.43 ± 10.79
STAR Total (*p*)	E	79.88 ± 12.34	84.87 ± 11.76
	C	79.69 ± 12.12	79.65 ± 11.74
STAR A (*n*)	E	80.37 ± 10.18	84.63 ± 8.91
	C	79.53 ± 9.09	79.73 ± 8.47
STAR AM (*n*)	E	84.70 ± 7.99	87.93 ± 8.71
	C	83.60 ± 8.46	83.83 ± 8.02
STAR M (*n*)	E	84.70 ± 9.30	88.97 ± 9.48
	C	82.87 ± 9.99	82.50 ± 9.90
STAR PM (*n*)	E	84.23 ± 11.45	90.80 ± 11.23
	C	81.80 ± 10.05	80.47 ± 10.98
STAR P (*n*)	E	80.80 ± 18.08	88.30 ± 14.83
	C	76.23 ± 12.60	76.40 ± 11.76
STAR PL (*n*)	E	79.57 ± 13.76	85.37 ± 13.25
	C	79.93 ± 13.32	78.73 ± 13.31
STAR L (*n*)	E	69.73 ± 12.70	75.87 ± 12.42
	C	70.70 ± 12.55	71.30 ± 11.63
STAR AL (*n*)	E	75.77 ± 11.06	79.80 ± 11.10
	C	76.17 ± 11.15	76.10 ± 10.18
STAR Total (*n*)	E	79.98 ± 10.81	85.21 ± 10.00
	C	78.85 ± 9.73	78.63 ± 9.20

Values are presented as mean ± SD.

E, experimental group; C, control group; p, preferred limb; n, non-preferred limb; STAR, Star Excursion Balance Test; A, anterior; AM, anteromedial; M, medial; PM, posteromedial; P, posterior; PL, posterolateral; L, lateral; AL, anterolateral.

### 3.5 Dynamic balance (between-group effects)

Group × time interaction analysis (Table 5) revealed significant between-group differences in several STAR variables, indicating superior improvements in the experimental group. Significant interactions were observed for posteromedial reach in both limbs (preferred: F = 9.038, *p* = 0.008; non-preferred: F = 7.385, *p* = 0.014), posterolateral reach (preferred: *p* = 0.042; non-preferred: *p* = 0.019), posterior reach (*p* = 0.038), and total reach distance (preferred: *p* = 0.015; non-preferred: *p* = 0.032). Effect sizes ranged from small to large (d = 0.30–0.81). No significant interactions were observed for anterior or lateral directions in most comparisons (*p* > 0.05), although the non-preferred anterior reach approached significance (*p* = 0.053). A borderline significant group × time interaction was also observed for the anterior reach in the preferred limb (*p* = 0.050), indicating a trend toward greater improvement in the experimental group compared with the control group; however, this finding did not reach the conventional threshold for statistical significance and should therefore be interpreted with caution.

**Table 5:** Group × time interaction effects for dynamic balance outcomes (star excursion balance test; repeated-measures ANOVA).

Variable	*F*	*P*	Cohen's d
STAR A (*p*)	4.426	0.050	0.29
STAR AM (*p*)	0.290	0.597	0.14
STAR M (*p*)	2.680	0.119	0.53
STAR PM (*p*)	9.038	0.008	0.81
STAR P (*p*)	5.008	0.038	0.30
STAR PL (*p*)	4.816	0.042	0.43
STAR L (*p*)	3.460	0.079	0.31
STAR AL (*p*)	0.348	0.348	0.18
STAR Total (*p*)	7.248	0.015	0.40
STAR A (*n*)	4.274	0.053	0.42
STAR AM (*n*)	1.945	0.180	0.40
STAR M (*n*)	3.171	0.092	0.46
STAR PM (*n*)	7.385	0.014	0.57
STAR P (*n*)	3.254	0.088	0.41
STAR PL (*n*)	6.599	0.019	0.42
STAR L (*n*)	2.752	0.114	0.48
STAR AL (*n*)	4.315	0.052	0.36
STAR Total (*n*)	5.379	0.032	0.48

Values represent group × time interaction effects obtained from repeated-measures ANOVA comparing experimental and control groups across pre- and post-intervention assessments.

Cohen's d represents effect size calculated for the experimental group. Positive values indicate greater improvements in the experimental group compared to the control group.

Statistical significance was set at *p* < 0.05.

STAR, Star Excursion Balance Test; p, preferred limb; n, non-preferred limb; A, anterior; AM, anteromedial; M, medial; PM, posteromedial; P, posterior; PL, posterolateral; L, lateral; AL, anterolateral.

### 3.6 Agility performance

Agility performance outcomes (SMSS) are summarized in Table 2, with between-group effects presented in Table 3. No statistically significant group × time interactions were found for SMSS performance in either limb condition (preferred: *p* = 0.051; non-preferred: *p* = 0.467). Although the preferred limb condition approached the threshold for significance, the overall results do not support a statistically meaningful training effect on agility.

### 3.7 Summary of findings

Overall, the sensorimotor training program induced significant improvements in selected motor control and dynamic balance variables, particularly in knee joint position sense at 60° flexion and in posteromedial and posterolateral STAR directions. Effect sizes ranged from small to large depending on the variable, indicating heterogeneous but consistent beneficial effects. No clear effects were observed for agility performance. Body mass and BMI remained stable throughout the intervention period. Full statistical details are reported in Tables 2–5 and Supplementary Tables S3, S4.

## 4 Discussion

### 4.1 Principal findings

The main finding of this study is that a five-week sensorimotor training program induced significant improvements in selected measures of dynamic balance and knee joint position sense in male handball players with hearing impairment. In contrast, no clear improvements were observed in agility performance. These findings are in line with previous research demonstrating that neuromuscular and sensorimotor training can improve postural control and proprioceptive function in team sport athletes (8–11, 38). These results are additionally supported by evidence showing that sensorimotor training enhances neuromuscular efficiency through improved afferent input and sensorimotor integration from muscle and joint mechanoreceptors (39), leading to more stable motor output (24, 25, 39). In populations with altered sensory input, such as individuals with hearing impairment, compensatory sensory reweighting may further enhance reliance on somatosensory feedback, amplifying training responsiveness (4, 29).

### 4.2 Dynamic balance adaptations

Significant improvements in several SEBT directions, particularly in posterior and posteromedial reaches, suggest enhanced dynamic postural control following sensorimotor training. These adaptations may be explained by improved neuromuscular coordination and increased efficiency of sensorimotor integration processes, which are commonly reported following balance-based training interventions (8, 9, 40). Similar improvements in dynamic balance after neuromuscular training programs in handball players have been previously reported (10, 11). The observed adaptations are consistent with findings from the broader literature showing that balance training has been associated with improvements in neuromuscular control, proprioception, and movement coordination (41, 42). In addition, auditory deprivation may lead to adaptive changes in multisensory integration, shifting postural control toward increased somatosensory and vestibular dependence (3, 28, 29). The observed improvements may also reflect task-specific adaptations, as SEBT performance is strongly influenced by lower limb control, hip stability, and intersegmental coordination (8, 9). From a practical standpoint, coaches should emphasize multidirectional, unilateral and unstable balance tasks to enhance dynamic postural control in athletes with hearing impairment.

### 4.3 Knee joint position sense and proprioception

Significant improvements in knee joint position sense at 60° flexion in both limbs suggest angle-specific proprioceptive adaptation. This finding is consistent with previous studies showing that proprioceptive acuity can be improved through targeted training interventions, particularly at mid-range joint angles where mechanoreceptor sensitivity is high (35, 43, 44). Similar improvements in proprioception following neuromuscular training have also been documented in team sport athletes, including handball players (11, 37, 44), as well as in studies examining sport-specific influences on knee joint position sense (45). These adaptations are consistent with enhanced afferent signal processing and improved central integration within sensorimotor networks (39, 44, 46). In individuals with hearing impairment, reduced auditory input may increase reliance on somatosensory feedback, potentially strengthening proprioceptive adaptation mechanisms during training (5, 29, 30). This aligns with the concept of sensory reweighting, where the central nervous system adapts by increasing the weighting of available sensory inputs. From a practical standpoint, training should include controlled knee flexion tasks around ∼60° to optimize proprioceptive adaptation and joint stability.

### 4.4 Agility performance

No significant improvements were observed in agility performance following the intervention. This finding suggests that short-duration sensorimotor training may not be sufficient to induce measurable changes in complex sport-specific motor tasks. Agility is a multifactorial ability influenced by strength, power, technique, and perceptual-cognitive components, which typically require longer and more specific training stimuli to improve (18, 21–23, 47). Similar findings have been reported in studies where balance-focused interventions improved postural control and coordination but showed limited transfer to sport-specific performance outcomes in trained athletes (10, 11, 48). This is consistent with literature suggesting limited transfer of isolated sensorimotor adaptations to high-intensity change-of-direction tasks in well-trained populations (2, 19, 49). The lack of significant change may also be explained by relatively high baseline performance levels in the studied cohort, limiting further adaptation potential. From a practical perspective, agility development in trained handball players requires longer interventions combined with reactive and sport-specific decision-making drills.

### 4.5 Practical implications

Sensorimotor training can be considered a useful complementary method in the preparation of athletes with hearing impairment, particularly for enhancing balance control and proprioceptive accuracy. Exercises involving neuromuscular control–oriented tasks and sport-specific movement patterns may be beneficial for improving motor control and reducing asymmetries (8, 11, 38). These adaptations may be relevant for injury prevention strategies in handball, where lower limb injuries are common and neuromuscular training has been shown to reduce injury risk in team handball players (7, 10, 11). Evidence from injury prevention literature suggests that improved neuromuscular control contributes to reduced risk of non-contact knee injuries (22, 23).

### 4.6 Limitations

Several limitations should be acknowledged. The relatively small sample size limits the generalizability of the findings and should be considered when interpreting the results. No *a priori* sample size calculation was performed before participant recruitment because of the limited availability of eligible elite handball players with hearing impairment. Consequently, the sample size was determined by the availability of athletes who met the predefined inclusion criteria during the recruitment period, which should be considered when interpreting the robustness of the findings. The intervention duration was relatively short, which may have been insufficient to induce broader performance adaptations, such as improvements in agility. No long-term follow-up assessment was conducted; therefore, the persistence of the observed adaptations over time remains unknown. Furthermore, no direct injury outcomes were measured, and conclusions regarding injury prevention therefore remain speculative. In addition, more advanced biomechanical or neurophysiological assessments (e.g., center of pressure dynamics or reflex responses) were not included, which could have provided deeper insight into the underlying mechanisms of adaptation. A *post hoc* power analysis indicated an achieved statistical power of 0.65 for the primary outcome analysis. Although significant intervention effects were observed for several variables, this suggests that the study may have been underpowered to detect smaller effects. Accordingly, non-significant findings should be interpreted with caution in the context of the sample size and study design.

### 4.7 Conclusion of discussion

Overall, the findings suggest that sensorimotor training may improve dynamic balance and knee proprioception in male handball players with hearing impairment, while agility improvements likely require longer or more specific training exposure. These results support the role of sensorimotor training as an effective neuromuscular intervention, particularly in populations with altered sensory integration strategies, such as athletes with hearing impairment.

## 5 Conclusions

The results suggest that sensorimotor training may improve dynamic balance and knee proprioception in male handball players with hearing impairment, while improvements in agility likely require a longer intervention duration. These findings indicate that targeted sensorimotor exercises may contribute to enhanced neuromuscular control in this population, particularly in components related to postural stability and proprioceptive function. Sensorimotor training may therefore be considered as a complementary method within training programs for athletes with hearing impairment; however, its effects on more complex performance outcomes such as agility appear to require further investigation with longer training exposure.

## Data Availability

The raw data supporting the conclusions of this article will be made available by the authors, without undue reservation.
