# Supplementary material for: Effects of a sensorimotor training program on balance and knee motor control in male handball players with hearing impairment: a randomized controlled trial
Source: Front Sports Act Living. 2026 Jul 29;8:1812503. doi: 10.3389/fspor.2026.1812503 (PMC13462459; doi:10.3389/fspor.2026.1812503)
Supplement: Supplementary file 1 [file Supplementaryfile1.docx]

Supplementary Material

# 1 Supplementary Figures and Tables

**Supplementary Table 1. Normality assessment (Shapiro–Wilk test)**

| Variable | Group | W | p |
| --- | --- | --- | --- |
| SMSS (p) | E | 0.89 | 0.19 |
| SMSS (p) | C | 0.86 | 0.08 |
| SMSS (n) | E | 0.91 | 0.31 |
| SMSS (n) | C | 0.99 | 1 |
| Ō 15° (p) | E | 0.96 | 0.89 |
| Ō 15° (p) | C | 0.86 | 0.072 |
| Ō 15° (n) | E | 0.9 | 0.21 |
| Ō 15° (n) | C | 0.93 | 0.46 |
| Ō 60° (p) | E | 0.9 | 0.25 |
| Ō 60° (p) | C | 0.87 | 0.11 |
| Ō 60° (n) | E | 0.97 | 0.92 |
| Ō 60° (n) | C | 0.98 | 0.97 |
| Ō 105° (p) | E | 0.93 | 0.43 |
| Ō 105° (p) | C | 0.86 | 0.088 |
| Ō 105° (n) | E | 0.93 | 0.47 |
| Ō 105° (n) | C | 0.94 | 0.56 |
| STAR Total (p) | E | 0.93 | 0.52 |
| STAR Total (p) | C | 0.97 | 0.97 |
| STAR Total (n) | E | 0.85 | 0.06 |
| STAR Total (n) | C | 0.87 | 0.11 |

Footnote: Values represent Shapiro–Wilk test statistics (W) and associated p-values for assessment of normality of distribution within each group. SMSS = speed of movement in defensive sliding task. Ō = absolute angular error in knee joint position sense. STAR = Star Excursion Balance Test composite reach scores expressed as percentage of leg length. Total = composite score across all directions. E, experimental group; C, control group; p, preferred limb; n, non-preferred limb. Statistical significance was set at p < 0.05.

**Supplementary Table 2. Homogeneity of variance (Levene’s test on change scores)**

| Variable | Comparison | F | p |
| --- | --- | --- | --- |
| SMSS (p) | E vs C | 0.006 | 0.94 |
| SMSS (n) | E vs C | 15.65 | 0.00093 |
| Ō 15° (p) | E vs C | 10.48 | 0.0046 |
| Ō 15° (n) | E vs C | 10.68 | 0.004 |
| Ō 60° (p) | E vs C | 11.27 | 0.0035 |
| Ō 60° (n) | E vs C | 14.77 | 0.0012 |
| Ō 105° (p) | E vs C | 8.8 | 0.0083 |
| Ō 105° (n) | E vs C | 13.86 | 0.0016 |
| STAR Total (p) | E vs C | 7.59 | 0.01 |
| STAR Total (n) | E vs C | 4.81 | 0.04 |

Footnote:Values represent Levene’s test for equality of variances performed on change scores (post–pre differences) between experimental (E) and control (C) groups. SMSS = speed of movement in defensive sliding task. Ō = absolute angular error in knee joint position sense. STAR = Star Excursion Balance Test composite reach scores expressed as percentage of leg length. Total = composite score across all directions. p, preferred limb; n, non-preferred limb. Statistical significance was set at p < 0.05.

**Supplementary Table 3. Within-group post hoc comparisons and effect sizes for motor control outcomes.**

| Variable | Experimental group Pre | Experimental group Post | p-value | Cohen's d | Control group Pre | Control group Post | p-value | Cohen's d |
| --- | --- | --- | --- | --- | --- | --- | --- | --- |
| SMSS (p) | 7.54 ± 0.69 | 7.39 ± 0.61 | 0.18 | 0.22 | 7.54 ± 0.66 | 7.68 ± 0.69 | 0.16 | (-)0.21 |
| SMSS (n) | 7.60 ± 0.71 | 7.45 ± 0.70 | 0.14 | 0.21 | 7.76 ± 0.78 | 7.69 ± 0.80 | 0.33 | 0.09 |
| Ō 15° (p) | 4.72 ± 2.31 | 3.92 ± 2.25 | 0.26 | 0.35 | 5.20 ± 3.38 | 5.88 ± 3.29 | 0.16 | (-)0.20 |
| Ō 60° (p) | 7.69 ± 4.61 | 4.32 ± 1.29 | 0.05 | 0.73 | 5.54 ± 3.96 | 5.53 ± 3.97 | 0.83 | 0.00 |
| Ō 105° (p) | 8.66 ± 5.21 | 7.72 ± 5.09 | 0.71 | 0.18 | 7.47 ± 3.45 | 6.74 ± 3.62 | 0.30 | 0.21 |
| Ō 15° (n) | 4.64 ± 2.61 | 4.42 ± 2.59 | 0.67 | 0.08 | 5.11 ± 3.01 | 4.42 ± 2.67 | 0.32 | 0.23 |
| Ō 60° (n) | 6.95 ± 4.07 | 3.78 ± 1.95 | 0.05 | 0.78 | 5.72 ± 3.95 | 5.76 ± 3.89 | 0.94 | (-)0.01 |
| Ō 105° (n) | 9.97 ± 4.15 | 9.57 ± 4.97 | 0.85 | 0.10 | 8.13 ± 4.46 | 8.16 ± 4.42 | 0.56 | (-)0.01 |

Footnote: Data are presented as mean ± SD. SMSS = speed of movement in defensive sliding task. Ō = absolute angular error in knee joint position sense. p = preferred limb; n = non-preferred limb. Pre = pre-intervention; Post = post-intervention. Within-group p-values were obtained using paired-samples t-tests. Cohen's d represents within-group effect size. Positive values of Cohen's d indicate improvement in performance, whereas negative values indicate a decrease in performance.

**Supplementary Table 4. Within-group post hoc comparisons and effect sizes for dynamic balance outcomes.**

| Variable | Experimental group Pre | Experimental group Post | p-value | Cohen's d | Control group Pre | Control group Post | p-value | Cohen's d |
| --- | --- | --- | --- | --- | --- | --- | --- | --- |
| STAR A (p) | 80.30 ± 14.59 | 84.60 ± 10.41 | 0.05 | 0.29 | 82.23 ± 13.68 | 82.33 ± 13.02 | 0.82 | 0.01 |
| STAR AM (p) | 82.47 ± 9.79 | 83.83. ± 10.21 | 0.60 | 0.14 | 81.67 ± 9.71 | 81.63 ± 8.50 | 0.96 | (-)0.00 |
| STAR M (p) | 84.17± 11.35 | 90.20 ± 15.57 | 0.15 | 0.53 | 83.67 ± 11.05 | 83.43 ± 10.94 | 0.44 | (-)0.02 |
| STAR PM (p) | 83.33 ± 10.90 | 92.20 ± 14.82 | 0.02 | 0.81 | 83.57 ± 12.85 | 83.07 ± 12.88 | 0.24 | (-)0.02 |
| STAR P (p) | 86.33 ± 17.73 | 91.60 ± 16.21 | 0.06 | 0.30 | 82.63 ± 15.95 | 82.23 ± 16.44 | 0.57 | (-)0.03 |
| STAR PL (p) | 79.33 ± 16.62 | 86.40 ± 12.96 | 0.04 | 0.43 | 80.00 ± 17.67 | 80.47 ± 17.95 | 0.40 | 0.03 |
| STAR L (p) | 68.27 ± 16.04 | 73.30 ± 13.39 | 0.09 | 0.31 | 68.57 ± 15.44 | 68.60 ± 14.46 | 0.95 | 0.00 |
| STAR AL (p) | 74.80 ± 11.47 | 76.83 ± 10.29 | 0.29 | 0.18 | 75.17 ± 11.26 | 75.43 ± 10.79 | 0.40 | 0.02 |
| STAR Total (p) | 79.88 ± 12.34 | 84.87 ± 11.76 | 0.02 | 0.40 | 79.69 ± 12.12 | 79.65 ± 11.74 | 0.87 | (-)0.00 |
| STAR A (n) | 80.37 ± 10.18 | 84.63 ± 8.91 | 0.05 | 0.42 | 79.53 ± 9.09 | 79.73 ± 8.47 | 0.64 | 0.02 |
| STAR AM (n) | 84.70 ± 7.99 | 87.93 ± 8.71 | 0.16 | 0.40 | 83.60 ± 8.46 | 83.83 ± 8.02 | 0.46 | 0.03 |
| STAR M (n) | 84.70 ± 9.30 | 88.97 ± 9.48 | 0.12 | 0.46 | 82.87 ± 9.99 | 82.50 ± 9.90 | 0.60 | (-)0.04 |
| STAR PM (n) | 84.23 ± 11.45 | 90.80 ± 11.23 | 0.04 | 0.57 | 81.80 ± 10.05 | 80.47 ± 10.98 | 0.09 | (-)0.13 |
| STAR P (n) | 80.80 ± 18.08 | 88.30 ± 14.83 | 0.10 | 0.41 | 76.23 ± 12.60 | 76.40 ± 11.76 | 0.61 | 0.01 |
| STAR PL (n) | 79.57 ± 13.76 | 85.37 ± 13.25 | 0.05 | 0.42 | 79.93 ± 13.32 | 78.73 ± 13.31 | 0.18 | (-)0.1 |
| STAR L (n) | 69.73 ± 12.70 | 75.87 ± 12.42 | 0.09 | 0.48 | 70.70 ± 12.55 | 71.30 ± 11.63 | 0.46 | 0.05 |
| STAR AL (n) | 75.77 ± 11.06 | 79.80 ± 11.10 | 0.06 | 0.36 | 76.17 ± 11.15 | 76.10 ± 10.18 | 0.93 | (-)0.01 |
| STAR Total (n) | 79.98 ± 10.81 | 85.21 ± 10.00 | 0.05 | 0.48 | 78.85 ± 9.73 | 78.63 ± 9.20 | 0.46 | (-)0.02 |

Footnote: Data are presented as mean ± SD. STAR = Star Excursion Balance Test composite reach scores expressed as percentage of leg length. A, anterior; AM, anteromedial; M, medial; PM, posteromedial; P, posterior; PL, posterolateral; L, lateral; AL, anterolateral; Total = composite score across all directions. p = preferred limb; n = non-preferred limb. Pre = pre-intervention; Post = post-intervention. Within-group p-values were obtained using paired-samples t-tests. Cohen's d represents within-group effect size. Positive values of Cohen's d indicate improvement in performance, whereas negative values indicate a decrease in performance.
